# Supplementary material for: Time-series transcriptome analysis identified differentially expressed genes in broiler chicken infected with mixed Eimeria species
Source: Front Genet. 2022 Aug 8;13:886781. doi: 10.3389/fgene.2022.886781 (PMC9393255; doi:10.3389/fgene.2022.886781)
Supplement: Supplementary file 2 [file DataSheet1.ZIP › 4dpi_KEGG.Gsea.1625100298584/KEGG_STEROID_BIOSYNTHESIS.html]

Details for gene set KEGG\_STEROID\_BIOSYNTHESIS[GSEA]

|  || Dataset | TMM\_4dpi\_gct\_format\_4dpi\_gct\_format.Class\_4dpi.cls #PC\_versus\_NC.Class\_4dpi.cls #PC\_versus\_NC\_repos |
| Phenotype | Class\_4dpi.cls#PC\_versus\_NC\_repos |
| Upregulated in class | 1 |
| GeneSet | KEGG\_STEROID\_BIOSYNTHESIS |
| Enrichment Score (ES) | 0.96608055 |
| Normalized Enrichment Score (NES) | 2.3870344 |
| Nominal p-value | 0.0 |
| FDR q-value | 0.0 |
| FWER p-Value | 0.0 |
Table: GSEA Results Summary

  

Fig 1: Enrichment plot: KEGG\_STEROID\_BIOSYNTHESIS      
 Profile of the Running ES Score & Positions of GeneSet Members on the Rank Ordered List

  

| SYMBOL | TITLE | RANK IN GENE LIST | RANK METRIC SCORE | RUNNING ES | CORE ENRICHMENT || 1 | DHCR7 | na | 17 | 2.286 | 0.1211 | Yes |
| 2 | MSMO1 | na | 25 | 2.193 | 0.2381 | Yes |
| 3 | CYP51A1 | na | 28 | 2.188 | 0.3552 | Yes |
| 4 | FDFT1 | na | 56 | 1.986 | 0.4594 | Yes |
| 5 | SQLE | na | 92 | 1.678 | 0.5464 | Yes |
| 6 | LSS | na | 138 | 1.500 | 0.6230 | Yes |
| 7 | DHCR24 | na | 221 | 1.313 | 0.6865 | Yes |
| 8 | LIPA | na | 288 | 1.188 | 0.7447 | Yes |
| 9 | SOAT1 | na | 350 | 1.121 | 0.7997 | Yes |
| 10 | NSDHL | na | 369 | 1.104 | 0.8573 | Yes |
| 11 | HSD17B7 | na | 410 | 1.054 | 0.9105 | Yes |
| 12 | SC5D | na | 418 | 1.048 | 0.9661 | Yes |
Table: GSEA details [plain text format]

  

Fig 2: KEGG\_STEROID\_BIOSYNTHESIS      
 Blue-Pink O' Gram in the Space of the Analyzed GeneSet

  

Fig 3: KEGG\_STEROID\_BIOSYNTHESIS: Random ES distribution      
 Gene set null distribution of ES for **KEGG\_STEROID\_BIOSYNTHESIS**
